# A bivalent recombinant vaccine targeting the S1 protein induces neutralizing antibodies against both SARS‐CoV‐2 variants and wild‐type of the virus

**DOI:** 10.1002/mco2.72

**Published:** 2021-05-17

**Authors:** Cai He, Jingyun Yang, Xuemei He, Weiqi Hong, Hong Lei, Zimin Chen, Guobo Shen, Li Yang, Jiong Li, Zhenling Wang, Xiangrong Song, Wei Wang, Guangwen Lu, Xiawei Wei

**Affiliations:** ^1^ Laboratory of Aging Research and Cancer Drug Target State Key Laboratory of Biotherapy and Cancer Center, National Clinical Research Center for Geriatrics West China Hospital, Sichuan University Chengdu China; ^2^ WestVac Biopharma Co. Ltd. Chengdu China

**Keywords:** bivalent vaccine, recombinant protein, SARS‐CoV‐2, variant

## Abstract

The emerging variants of severe acute respiratory syndrome coronavirus‐2 (SARS‐CoV‐2) in pandemic call for the urgent development of universal corona virus disease 2019 (COVID‐19) vaccines which could be effective for both wild‐type SARS‐CoV‐2 and mutant strains. In the current study, we formulated protein subunit vaccines with AS03 adjuvant and recombinant proteins of S1 subunit of SARS‐CoV‐2 (S1‐WT) and S1 variant (K417N, E484K, N501Y, and D614G) subunit (S1‐Mut), and immunized transgenic mice that express human angiotensin‐converting enzyme 2 (hACE2). The S1 protein‐specific antibody production and the neutralization capability for SARS‐CoV‐2 and B.1.351 variant were measured after immunization in mice. The results revealed that the S1‐Mut antigens were more effective in inhibiting the receptor‐binding domain and ACE2 binding in B.1.351 variant than in wild‐type SARS‐CoV‐2. Furthermore, the development of a bivalent vaccine exhibited the ideal neutralization properties against wild‐type and B.1.351 variant, as well as other variants. Our findings may provide a rationale for the development of a bivalent recombinant vaccine targeting the S1 protein that can induce the neutralizing antibodies against both SARS‐CoV‐2 variants and wild‐type of the virus and may be of importance to explore the potential clinical use of bivalent recombinant vaccine in the future.

## Graphical abstract

The bivalent recombinant vaccine composed of S1 subunit of SARS‐CoV‐2 and S1 variant (K417N, E484K, N501Y, and D614G) subunit could induce the neutralizing antibodies against both SARS‐CoV‐2 variants and wild‐type of the virus.

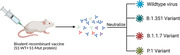

## 1 INTRODUCTION

The corona virus disease 2019 (COVID‐19) caused by severe acute respiratory syndrome coronavirus‐2 (SARS‐CoV‐2) has evolved into a pandemic and become a life‐threatening global problem.^1^, ^2^ SARS‐CoV‐2 consists of a positive‐sense, single‐stranded RNA genome, the inner nucleocapsid proteins, an outer envelope, and spike glycoprotein.^3^ The receptor‐binding domain (RBD) in the S1 subunit of spike protein mediated the recognition and binding of SARS‐CoV‐2 to the receptor angiotensin‐converting enzyme 2 (ACE2) on host cells.^4^ To date, over 140 million patients with COVID‐19 have been diagnosed worldwide. Comfortingly, with the understanding of SARS‐CoV‐2 and the accumulation of experiences in treating COVID‐19, recombinant neutralizing antibodies and kinds of vaccines have been developed, which bring hope and confidence to control and prevent the COVID‐19 pandemic.^5^, ^6^, ^7^ As of 14 April 2021, a total of 751,452,536 vaccine doses have been administered worldwide (https://covid19.who.int/). However, as the pandemic rages on, several variants have been reported, raising concerns that these variants might add fuel to the pandemic.

The main SARS‐CoV‐2 mutant strains, including B.1.1.7, B.1.351 (also known as 501Y.V2 or 20H), and B.1.1.248 (also known as P.1) have been reported. These mutations are mainly located in the spike protein. Previous studies have reported that mutations in spike protein, especially in S1 subunit including RBD, induced immune escape, changed the binding ability of the virus to ACE2 to increase the transmissibility and decreased efficacy of existing drugs and vaccines.^3^, ^8^ B.1.1.7 variant mainly burst in UK was resistant to some monoclonal antibodies (mAbs).^9^ In particular, B.1.351 variant as a dominant variant in South Africa, characterized by three amino acid mutations on the K417N, E484K, and N501Y in RBD accompanying with four substitutions and a deletion in the N‐terminal domain (NTD), decreased neutralization activity of antibodies induced by non‐B.1.351 SARS‐CoV‐2 infection or vaccination and increased transmissibility.^10^, ^11^, ^12^, ^13^ More importantly, the Novavax NVX‐CoV2373 subunit vaccine revealed a decreased efficacy from 89.3% to 49.4% in clinical studies in South Africa.^12^ And the efficacy of the ChAdOx1 chimpanzee adenoviral‐vectored vaccine (AZD1222) against B.1.351 was only 10.4%.^14^ Therefore, it is highly urgent to develop a universal coronavirus vaccine that is effective for both wild‐type SARS‐CoV‐2 and mutant strains to prevent the pandemic.

Mammalian cells expression system is a simple, rapid, inexpensive, and efficient method for protein expression.^15^, ^16^ In this study, we used protein subunit vaccines based on S1‐WT and S1‐Mut expressed by 293T cells, and evaluated their protective effects against pseudoviruses of wild‐type SARS‐CoV‐2 and variants. Furthermore, we used a bivalent vaccine formulated with S1‐WT and S1‐Mut recombinant proteins to estimate the cross‐protection against both wild and mutant strains of SARS‐CoV‐2. Our results laid the foundation for the development of vaccines against both wild‐type and variants of SARS‐CoV‐2.

## 2 RESULTS

### 2.1 Identification of antibodies against the RBD and S1 proteins

**FIGURE 1:**
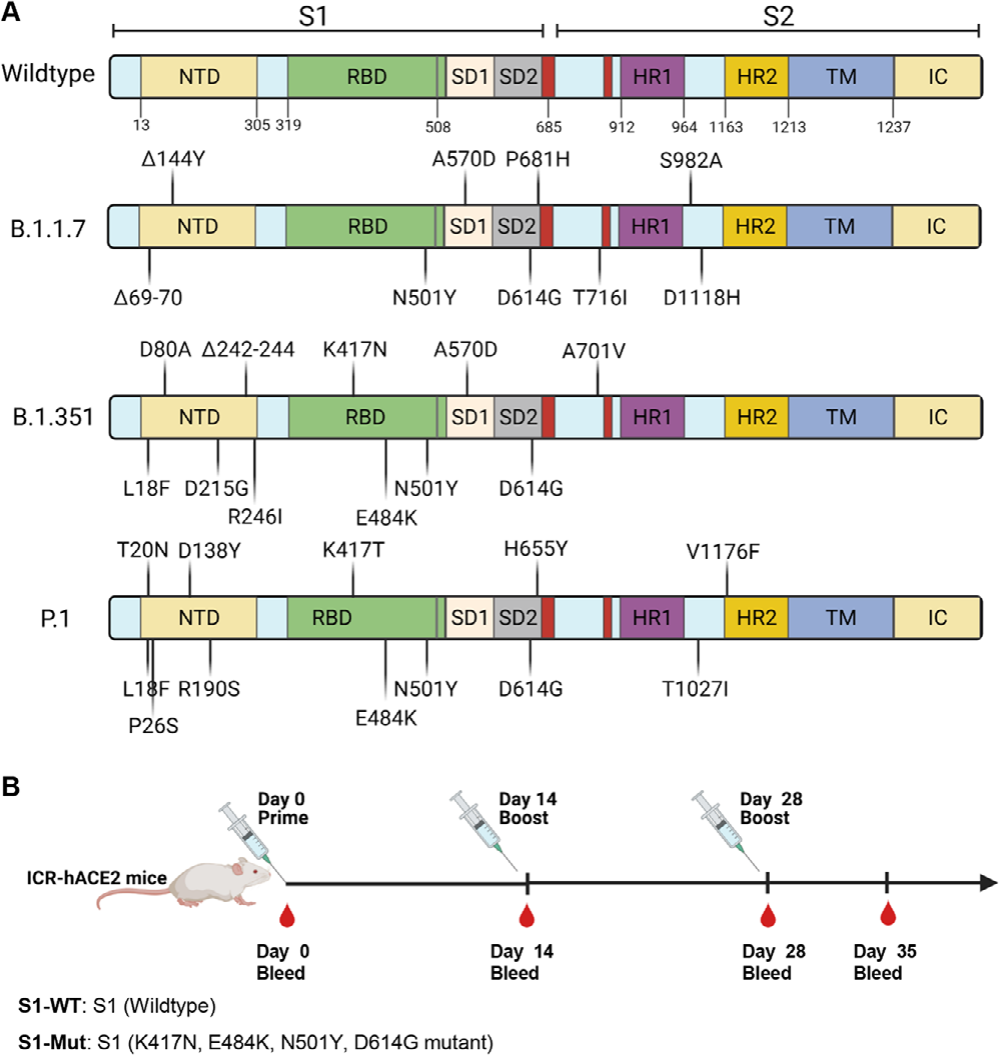
The summary of SARS‐CoV‐2 mutant virus strains and immunization schedule of S1‐WT and S1‐Mut proteins. (A) Mutations in the viral spike identified in B.1.1.7, B.1.351, and P.1. (B) The illustration of immunization and sampling schedule

We summarized the current main SARS‐CoV‐2 mutant strains, including B.1.1.7, B.1.351, and P.1 (Figure 1A). Based on D614G, other mutations existing in RBD are K417N/K417T, E484K, and N501Y which might affect the recognition and binding of SARS‐CoV‐2 to ACE2. Therefore, we chose S1‐Mut which contains K417N, E484K, N501Y, and D614G to formulate the recombinant protein vaccine.

**FIGURE 2:**
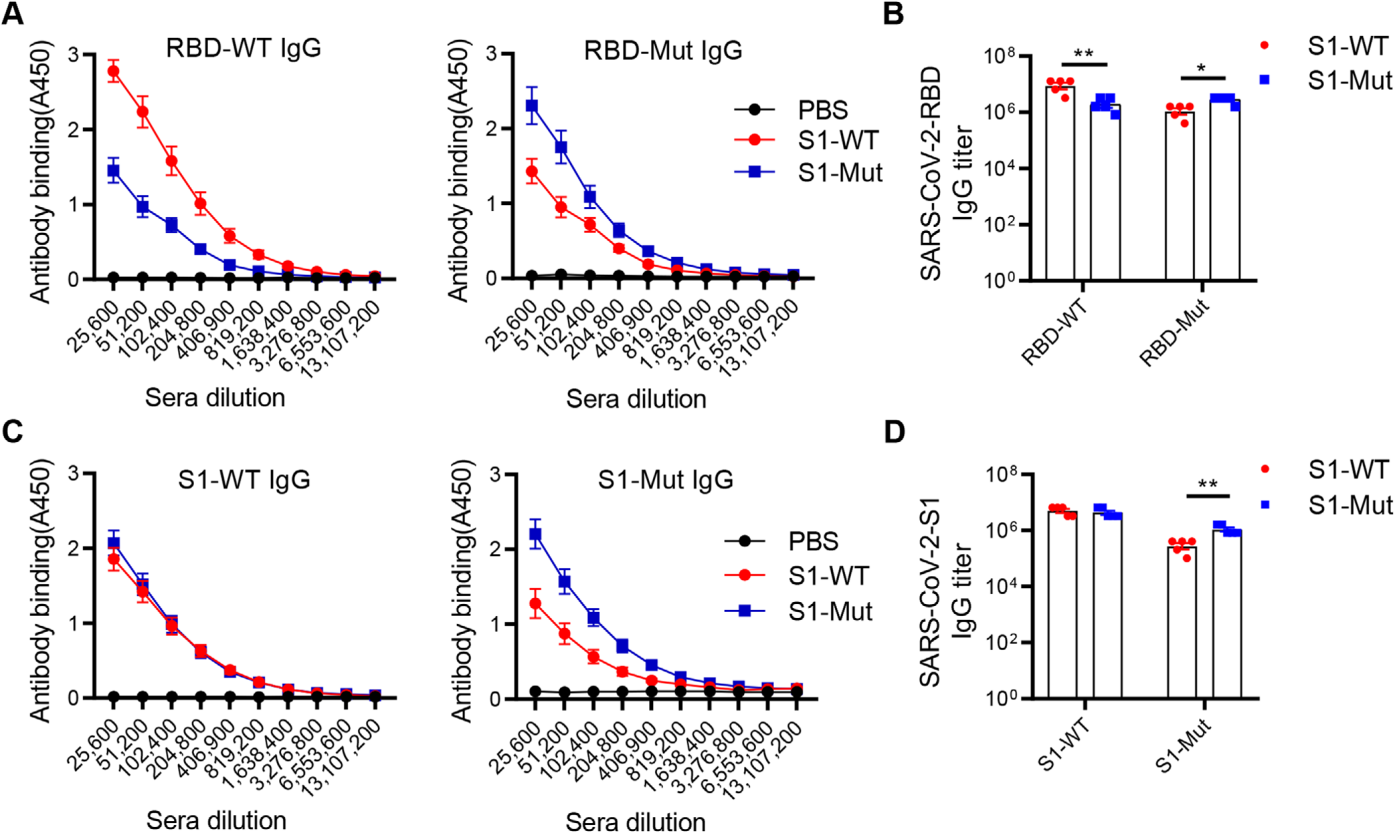
ICR‐hACE2 mice immunized with S1‐WT or S1‐Mut produced SARS‐CoV‐2 RBD‐ and S1‐specific antibodies. (A) Detection of RBD‐WT (Left) or RBD‐Mut (Right)‐specific IgG in series of diluted mouse sera. (B) The antibody titers of RBD‐WT or RBD‐Mut IgG in sera of mice immunized with S1‐WT or S1‐Mut proteins. (C) Detection of S1‐WT (Left) or S1‐Mut (Right)‐specific IgG in series of diluted mouse sera. (D) The antibody titers of S1‐WT or S1‐Mut IgG in serum of immunized mice. Serum antibody binding was measured as absorbance at 450 nm. Data are presented as mean ± SEM of five mouse sera per group. *p* values were determined by T‐text analysis. *p < 0.05, **p < *0.01*

To determine antibody response induced by two spike proteins, hACE2 mice with Institute of Cancer Research (ICR) background were intramuscularly vaccinated with S1‐WT or S1‐Mut proteins on day 0, 14, 28, respectively. Serum samples were collected before each immunization and 7 days after the last administration (Figure 1B). RBD‐WT, RBD‐Mut (K417N, E484Kand N501Y), S1‐WT, and S1‐Mut (K417N, E484K, N501Y, and D614G) proteins were used as coated antigens for antibodies assay. Consistent with our expectations, we observed that higher RBD‐WT‐specific IgG antibodies in S1‐WT‐immunized mice (Figure 2A), while S1‐Mut protein induced stronger IgG antibody responses against RBD‐Mut and S1‐Mut, and both of them elicited strong S1‐WT‐specific IgG responses (Figure 2C). The geometric mean titers (GMT) of RBD‐WT and RBD‐Mut‐specific antibodies in serum from S1‐WT group were 8.65 × 10^6^ and 1.08 × 10^6^, respectively. In S1‐Mut group, the GMT of RBD‐WT and RBD‐Mut were 1.88 × 10^6^ and 2.85 × 10^6^ (Figure 2B). Comparable GMT of S1‐WT IgG antibodies in serum was found between two groups; however, the GMT of S1‐Mut‐specific antibodies in serum from S1‐Mut group (1.08×10^6^) was much higher than that of S1‐WT group (2.7×10^5^) (Figure 2D). These results demonstrated that S1‐WT protein could elicit stronger wild‐type S1 and RBD‐specific antibody responses, and S1‐Mut protein could be a better immunogen for mutant S1 and RBD‐specific antibody responses.

### 2.2 Recombinant protein vaccines induced antibodies to inhibit the binding between RBD and ACE2

**FIGURE 3:**
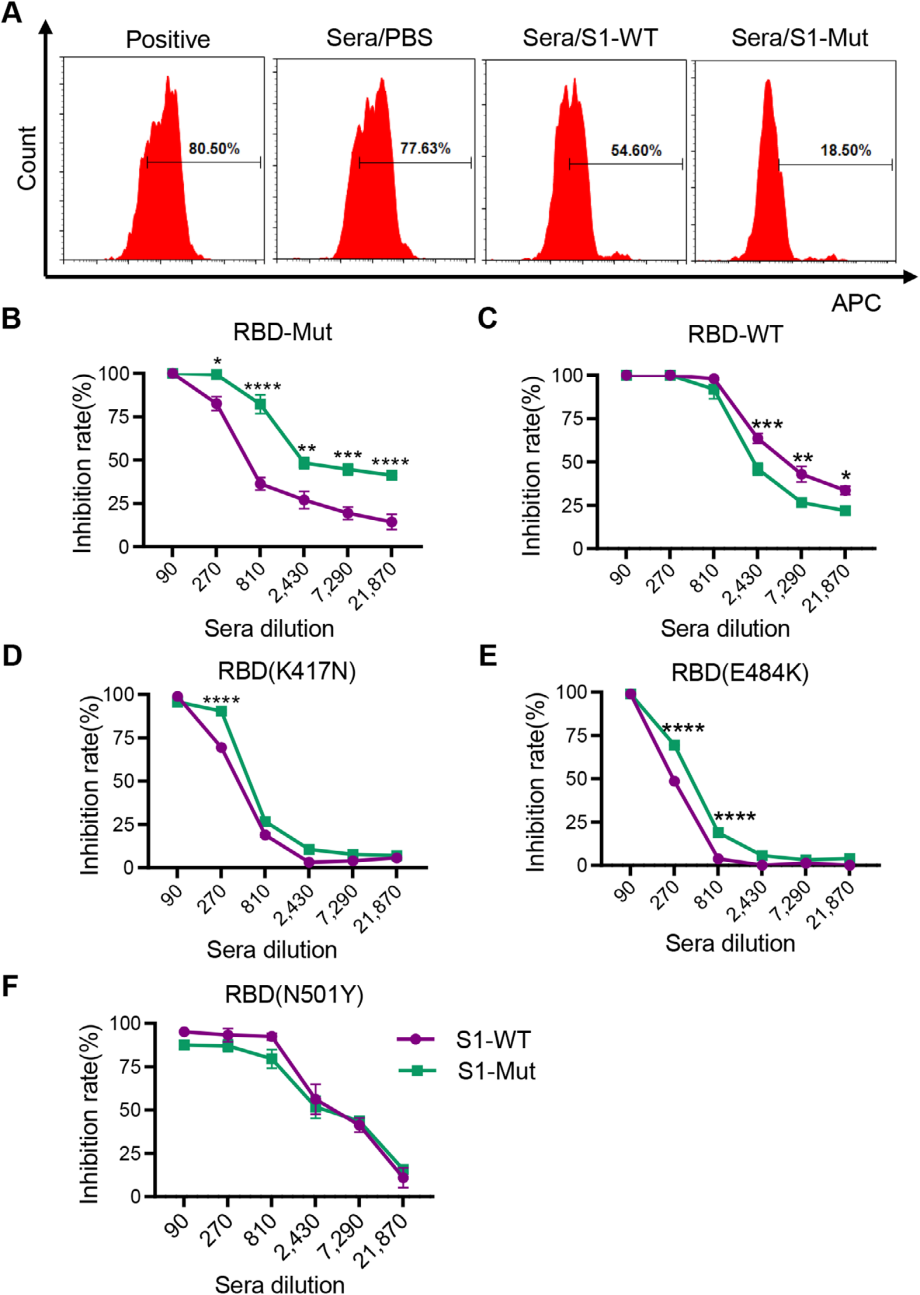
Sera from immunized mice with S1‐WT or S1‐Mut proteins blocked RBD binding to hACE2 receptor. (A) Representative graphs of flow cytometry represent blockade of RBD‐Mut binding to cell surface ACE2 receptor by immune sera. The ratio of sera to RBD‐Mut protein in flow fluorogram was 1:810. From left to right: Positive: without immune sera; PBS: sera from mice treated with PBS as a control; S1‐WT: sera from mice treated with S1‐WT protein; S1‐Mut: sear from mice treated with S1‐Mut protein. (B) Inhibition rate of RBD‐Mut binding to cell surface ACE2 receptor. (C‐F) Inhibition rate of RBD(WT), RBD(K417N), RBD(E484K), RBD(N501Y) binding to cell surface ACE2 receptor. Data are presented as mean ± SEM. *p* values were determined by two‐way analysis of variance (ANOVA). *p < 0.05, **p < *0.01*, ***p < *0.001*, ****p < *0.0001*

RBD‐WT and RBD‐Mut (K417N, E484K, N501Y) proteins were used for binding cell surface receptor ACE2. Note that 80.5% 293T/ACE2 cells were binding with RBD‐Mut in the absence of immune sera, which was used as a positive control. There was no inhibitory activity with sera from mice immunized with PBS, with the appearance of over 77.36% RBD‐Mut positive 293T/ACE2 cells. Remarkably, after incubated with serum from S1‐Mut immunized mice, only 18.5% cells were detected as positive. In contrast, 54.6% 293T/ACE2 cells were RBD‐Mut positive in the presence of serum from S1‐WT group (Figure 3A and 3B). For assay of RBD‐WT protein binding to ACE2, serum from mice vaccinated with S1‐WT showed much stronger blockade than S1‐Mut group (Figure 3C). To figure out which single mutation could cause the phenomenon, we used three mutant RBD proteins, including RBD (K417N), RBD (E484K), and RBD (N501Y). There was no significant difference in the ability to block RBD (K417N) and RBD (N501Y) between the two groups (Figures 3D and 3F). Interestingly, the serum of S1‐Mut group showed a better ability to inhibit RBD (E484K) than S1‐WT group (Figure 3E). These findings demonstrated that immunization with S1‐WT and S1‐Mut proteins has a stronger blockade on RBD‐WT and RBD‐Mut, respectively, and suggested that the E484K mutation might play an essential role in the resistance of S1‐WT.

### 2.3 Susceptibility of mutant or wild‐type pseudoviruses to neutralization by sera from mice immunized with S1‐WT or S1‐Mut

**FIGURE 4:**
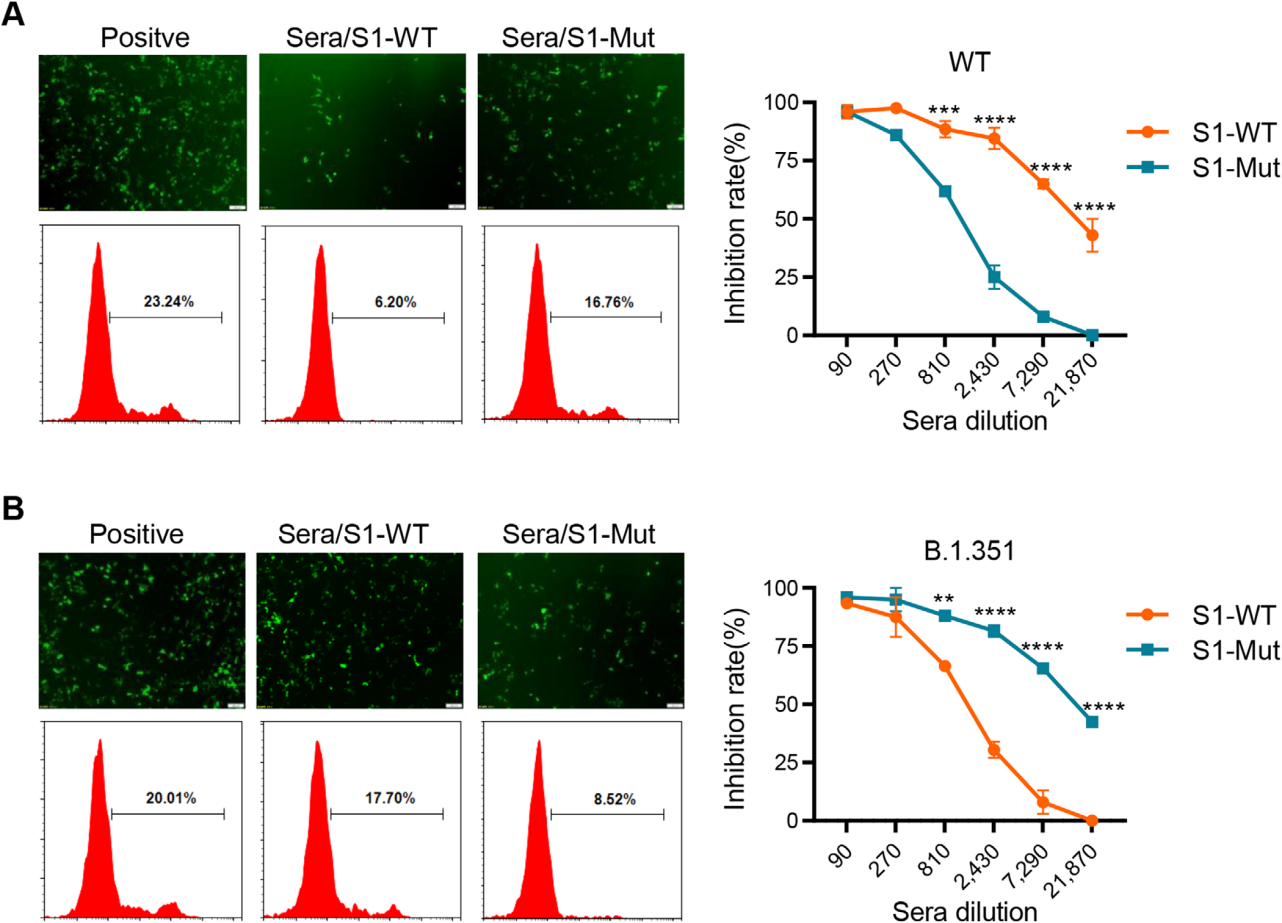
Sera from immunized mice blocked SARS‐CoV‐2 EGFP‐expressing pseudovirus infection into 293T/ACE2 cells. (A) Fluorescent images (Left) showed that sera from mice immunized with S1‐WT or S1‐Mut inhibited infectivity of wild‐type pseudovirus. Inhibition rate (Right) was calculated as described above. (B) Fluorescent images (Left) showed that sera from mice immunized with S1‐WT or S1‐Mut inhibited infectivity of B.1.351 pseudovirus. Inhibition rate (Right) was calculated as described in Methods. Cells infected by SARS‐CoV‐2 pseudovirus were recorded as EGFP positive. The dilution of sera in fluorescent images was at 1:2430. Scale bar, 100 μm. Data are presented as mean ± SEM. *p* values were determined by two‐way analysis of variance (ANOVA). **p < *0.01*, ***p < *0.001*, ****p < *0.0001*

We wonder if the neutralizing capabilities of immune sera were influenced by mutated pseudovirus. Therefore, we used two EGFP‐expressing pseudoviruses, wild‐type, and B.1.351 which is one of the most prevalent mutant strains of SARS‐CoV‐2 around the world. We observed that the number of EGFP‐expressing cells sharply decreased when WT pseudovirus was incubated with immune sera from mice immunized with S1‐WT at 1:2,430 dilution, but this blockade disappeared in the S1‐Mut group at the same dilution (Figure 4A). In contrast, sera from S1‐WT group almost loss the inhibitory ability against B.1.351 pseudovirus at 1:7,290 dilution, while sera from S1‐Mut group have more than 50% inhibition rate at the same dilution (Figure 4B).

**FIGURE 5:**
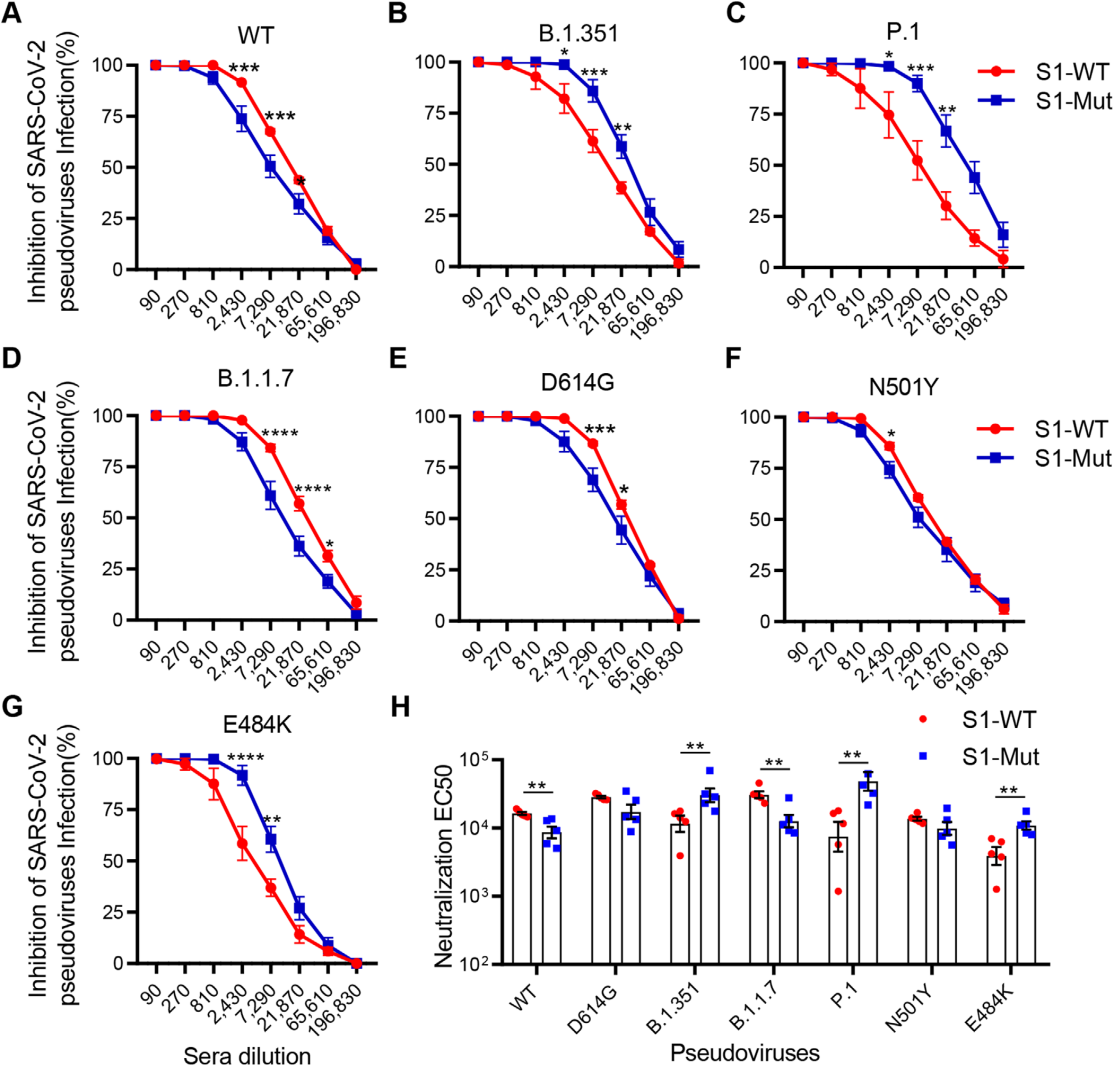
Sera from mice immunized with S1‐WT or S1‐Mut blocked SARS‐CoV‐2 luciferase‐expressing pseudovirus infection. (A‐G) Sera with series of dilution inhibited infection of pseudovirus with or without mutation (WT, B.1.351, P.1, B.1.1.7, D614G, N501Y, E484K). Data are Mean ± SEM. *p* values were determined by two‐way analysis of variance (ANOVA). (H) Neutralization EC50 is defined as the inverse dilution that achieved 50% neutralization, which was calculated by GraphPad Prism. Data are presented as mean ± SEM. *p* values were determined by T‐text. *p < 0.05, **p < *0.01*, ***p < *0.001*, ****p < *0.0001*

To further investigate the neutralizing effect of two spike proteins against other mutant pseudoviruses, wild‐type, D614G, B.1.1.7, B.1.351, and P.1 pseudoviruses with luciferase‐expressing were used. Consistent with other studies, the protective effect of serum from S1‐WT‐immunized mice against wild‐type, D614G, B.1.1.7 pseudoviruses did not obviously change (Figures 5A, 5D, 5E and 5H), but the protective effect against B.1.351 and P.1 significantly decreased (Figures 5B and 5C). Serum from mice immunized with S1‐Mut protein showed superior protection against B.1.351 and P.1 (Figures 5B and 5C), but its protection against wild‐type and B.1.1.7 was indeed reduced (Figure 5D). To understand the specific mutations responsible for the observed changes, we tested the sera from S1‐WT and S1‐Mut groups against pseudovirus containing only a single mutate site which was found in B.1.1.7, B.1.351, and P.1. The protective effect was not significantly different between sera from mice immunized with S1‐WT or S1‐Mut proteins against pseudovirus with N501Y (Figure 5F). However, the neutralizing effect of serum from S1‐WT group was impaired for pseudovirus with E484K mutation. Nevertheless, S1‐Mut protein showed a stronger neutralizing effect for E484K pseudovirus (Figure 5G). These findings suggest that S1‐Mut protein could induce stronger protective immunity to block mutant viruses containing E484K mutation, such as B.1.351 and P.1.

### 2.4 The bivalent vaccine showed excellent neutralization properties against various pseudoviruses

**FIGURE 6:**
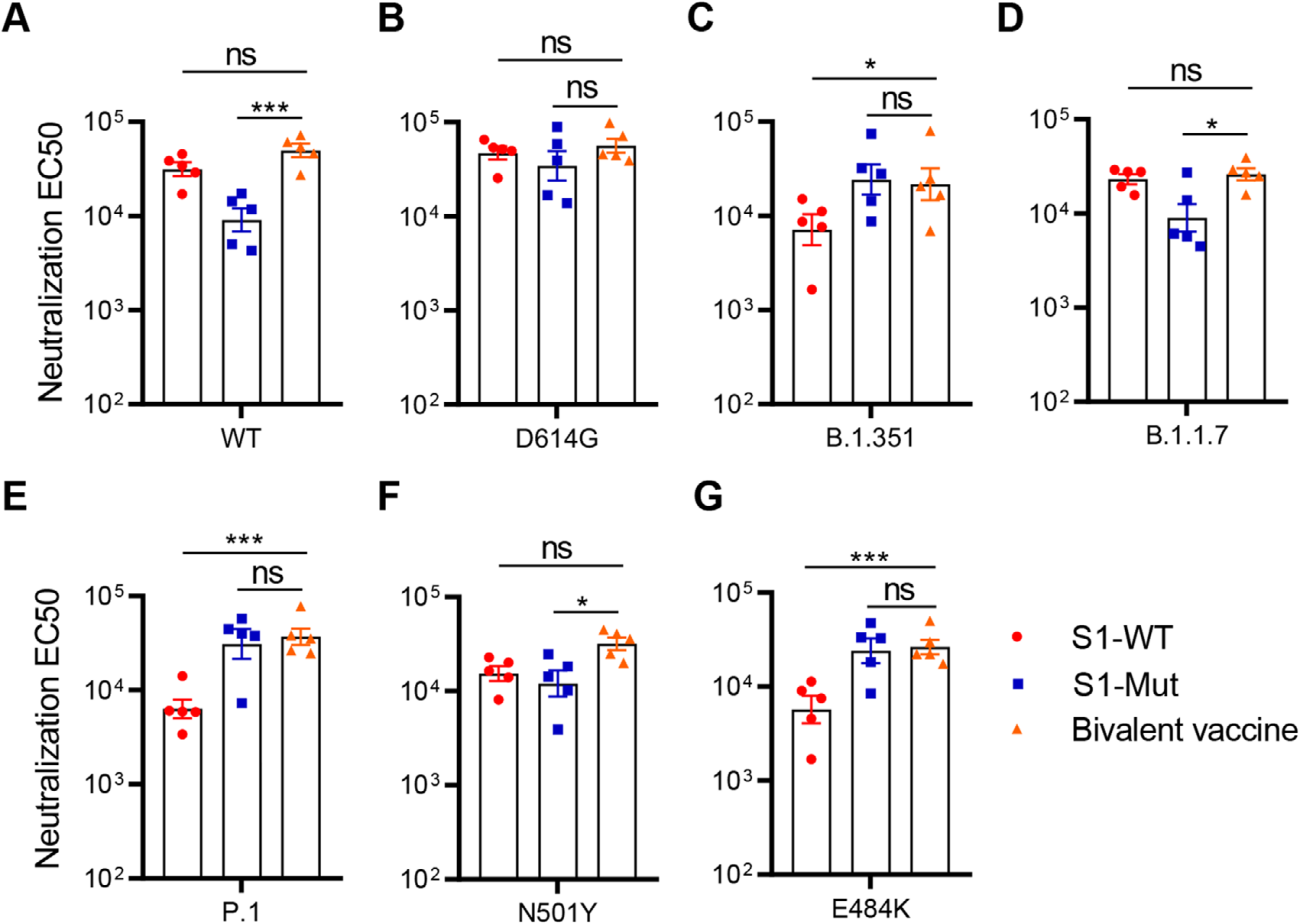
Sera from mice immunized with bivalent vaccine mixed with S1‐WT and S1‐Mut blocked a series of SARS‐CoV‐2 luciferase‐expressing pseudovirus infection. (A‐G) Neutralizing antibody titer of sera from mice immunized with S1‐WT, S1‐Mut, Bivalent vaccine. Neutralization EC50 is defined as the inverse dilution that achieved 50% neutralization. Data are presented as mean ± SEM. P values were determined by one‐way analysis of variance (ANOVA). *P < 0.05, ***P < *0.001*

Based on the results mentioned above, we speculated that combined immunization of these two spike proteins would exert strong protection against both wild and mutant strains of SARS‐CoV‐2. Therefore, we used AS03 as an adjuvant to immunize ICR‐hACE2 mice with S1‐WT protein (10 μg per mice), S1‐Mut protein (10 μg per mice), bivalent vaccine (5 μg S1‐WT and 5 μg S1‐Mut proteins per mice). The schedule of immunization and sampling was the same as described in Figure 1B. The results showed that the bivalent vaccine induced higher neutralization titer against WT and B.1.1.7 pseudoviruses compared with S1‐Mut vaccine, but no significant differences were observed in neutralization titer compared with S1‐WT vaccine (Figures 6A and 6B). Furthermore, the protective effect of the bivalent vaccine against B.1.351 and P.1 significantly increased compared to S1‐WT immunization. These results suggest that the bivalent vaccine showed excellent neutralization against wild‐type and B.1.351 pseudoviruses, and even against other variants (Figure 6).

### 2.5 Safety evaluation of bivalent vaccine in mice

**FIGURE 7:**
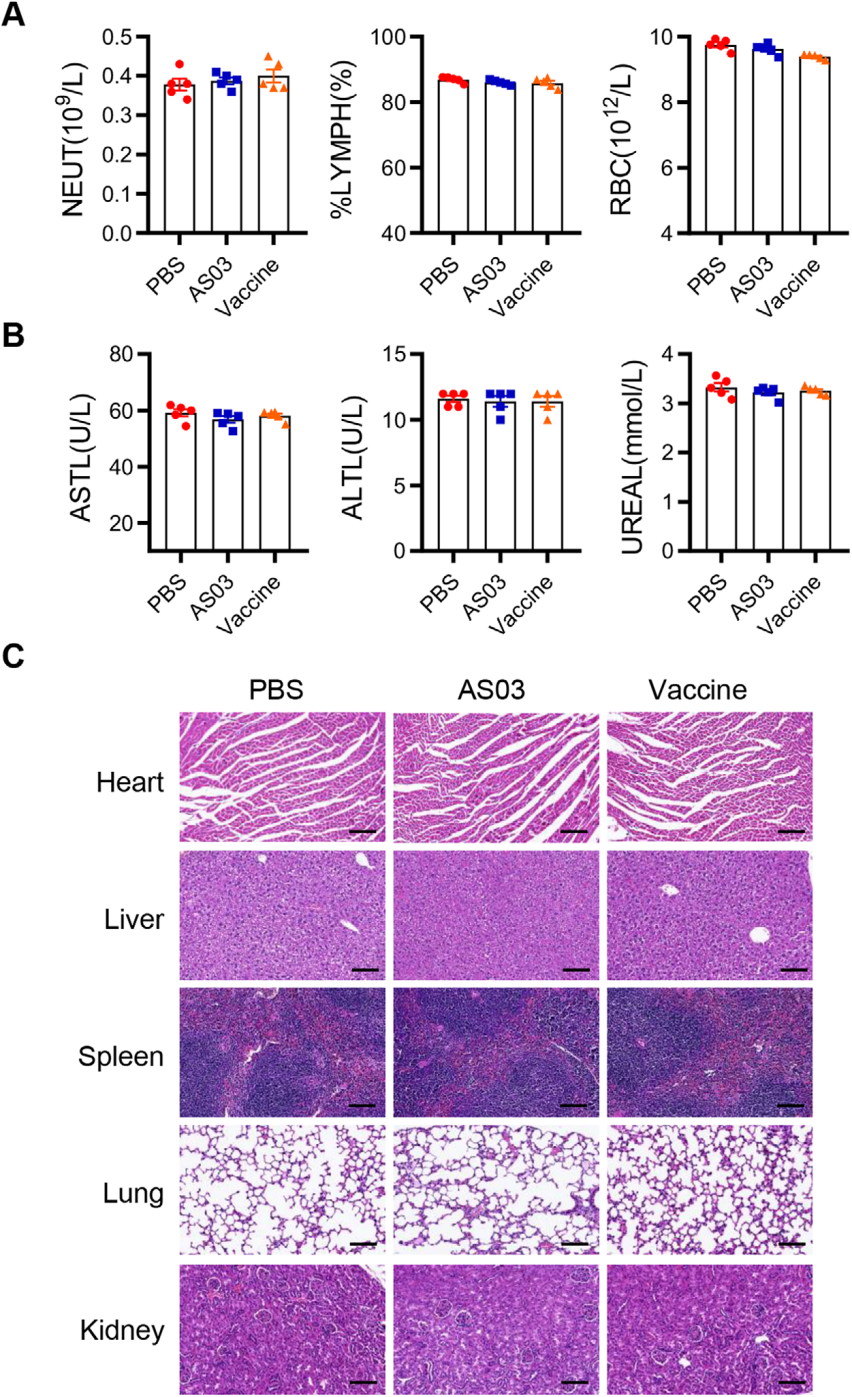
Safety evaluation of bivalent vaccine in mice. ICR‐hACE2 mice were immunized with PBS (PBS group), AS03 adjuvant (AS03 group) and the bivalent vaccine (vaccine group). (A and B) The peripheral blood counts (A) and biochemical indexes (B) in PBS, AS03 and bivalent vaccine groups. (C) Pathological H&E stain of various organs from immunized mice. Scale bar represents 100 μm

Next, we estimated the safety of the candidate bivalent vaccine in mice. There were no significant differences in body weight, skinfold, behavior change, and appetite among the PBS, AS03, and bivalent vaccine groups. And no adverse changes were observed in peripheral blood counts and differentials biochemical indexes such as ASTL, ALTL, etc. (Figures 7A and 7B). Besides, there were no pathological changes in heart, liver, spleen, lung, and kidney tissues among the PBS, AS03, and bivalent vaccine groups (Figure 7C).

## 3 DISCUSSION

The mutation on coronavirus might have changes on its transmission, disease, and vaccination effects. To date, the well‐recognized and threatening variants of SARS‐CoV‐2 include B.1.1.7, B.1.351, and P.1.^9^, ^17^ With the critical mutations in the region of spike protein, these variants resulted in increased transmissibility and impaired efficacy of vaccines. Notably, the B.1.351 variant is first identified in South Africa and became a dominant strain subsequently. Several studies have revealed that B.1.351 was less likely to be neutralized by convalescent plasma from COVID‐19 patients or plasma from people who received the vaccination of the COVID‐19 vaccine.^18^, ^19^ Some pharmaceutical manufacturers have announced the plans to investigate and test vaccines based on emerging variants.^20^ Therefore, there is an urgent need to explore universal protective vaccines against SARS‐CoV‐2 and mutant strains.

In the current study, several observations have been made concerned with the recombinant vaccines for inhibition of SARS‐CoV‐2 and B.1.351. After immunization in mice, S1‐WT protein elicited strong RBD‐WT‐specific IgG response, while S1‐Mut protein induced stronger RBD‐Mut and S1‐Mut IgG antibody responses; both elicited strong S1‐WT‐specific IgG response. Moreover, the neutralization antibodies induced by recombinant S1‐WT displayed strong blockade on RBD‐WT, while neutralization antibodies induced by S1‐Mut protein displayed stronger blockade on RBD (E484K) and RBD‐Mut, respectively. In addition, sera of S1‐Mut immunized mice could more effectively inhibit the B.1.351 pseudovirus while compared with the sera from S1‐WT group. Based on the results mentioned above, we optimized the vaccine with a combined immunization of two spike proteins, which resultantly exerted strong protection against both wild and mutant strains of SARS‐CoV‐2. In summary, we evaluated the protective effects of S1‐WT and S1‐Mut protein subunit vaccines against pseudoviruses of SARS‐CoV‐2 and B.1.351 variant.

Previous studies have reported that the mutation in the spike protein affected the immune response and vaccine efficacy, especially for RBD‐based vaccines.^3^, ^8^, ^17^, ^21^ Wang et al reported that B.1.351 variant is refractory to neutralization by most NTD mAbs and multiple individual mAbs to RBD, which might be due to the E484K mutation in the S1 subunit of RBD.^9^ Meanwhile, others discovered that the single E484K mutation impaired the binding ability of serum polyclonal neutralizing antibodies induced by previous SARS‐CoV‐2 strains infection or vaccines.^22^, ^23^ Consistent with these studies, our results showed that S1‐WT protein displayed lower neutralization titers against the mutant viruses containing E484K mutation, such as B.1.351 and P.1. Thrillingly, we discovered that the sera from mice immunized with S1‐Mut reduced the affinity between the ACE2 and RBD with E484K mutation, and S1‐Mut recombinant protein induced strong protective immunity to block B.1.351 and P.1 pseudoviruses containing E484K mutation. However, the S1‐Mut recombinant vaccine showed a poor protective role against previous SARS‐CoV‐2 strains without mutants in RBD. These results suggest that the monovalent recombinant protein vaccine could induce strong protective immunity against few strains but had limited protective efficacy against other strains. Based on these results, we combined the S1‐WT and S1‐Mut proteins to form a bivalent vaccine, and our bivalent vaccine showed cross‐protection against both wild type and variants of SARS‐CoV‐2, which could be a candidate as a universal vaccine against SARS‐CoV‐2 and its mutants. Besides, fusion protein with different antigens has been reported as a cross‐protective bivalent vaccine to prevent several other viruses.^24^, ^25^, ^26^ Therefore, we are delving into cross‐protective bivalent fusion vaccines composed of wild‐type and mutant spike protein fragments, which might be a promising strategy to prevent SARS‐CoV‐2 and mutant strains.

Taken together, our findings may provide a rationale for the development of a bivalent recombinant vaccine targeting the S1 protein that can induce the neutralizing antibodies against both SARS‐CoV‐2 variants and wild‐type of the virus, and may be of importance to explore the potential clinical use of bivalent recombinant vaccine in the future.

## 4 MATERIALS AND METHODS

### 4.1 Cell culture

293T cells were purchased from the American Type Culture Collection. 293T cells stably highly express ACE2 cells (293T/ACE2) were generated in our laboratory as previously reported.^27^ We cultured the cells in Dulbecco's modified Eagle's medium (DMEM, Gibco, USA) with 10% fetal bovine serum (FBS, PAN‐Biotech, Germany), 0.1 mg/ml streptomycin and 100U penicillin at 37°C with 5% CO_2_.

### 4.2 RBD protein expression and purification

The baculovirus‐insect cell expression system was used to produce wild‐type RBD (RBD‐WT) of spike as previously reported.^27^ In brief, the GP67‐Trx‐His‐EK‐RBD was transferred into the pFastBac1 vector by gene recombination technology, then transfect the bacmid into insect Sf9 cells with LipoInsect Transfection Regent (Beyotime Biotechnology). The GP67 signal peptide sequence was used to ensure the effective secretion of the protein, insect thioredoxin (TRX) to help the protein fold correctly and improve the stability of the antigen structure. After 72 h, we collected the supernatants containing the packaged recombinant baculoviruses and then passaged the baculovirus in Sf9 three times for protein production. For protein purification, the collected supernatants were passed through a 5‐ml HisTrap excel column, followed by a Superdex 200 Increase 10/300 GL column. Finally, we dissolved the protein in a buffer consisting of 20 mM Tris‐HCL and 150 mM NaCl. Recombinant RBD protein was used to detect specific antibodies.

### 4.3 Vaccine formulation and vaccinations of mice

Spike S1 recombinant protein (S1‐WT, aa:16‐685), spike S1 (K417N, E484K, N501Y, D614G) recombinant protein (S1‐Mut, aa:16‐685), RBD protein with Fc fragment (RBD‐WT, aa: 319–541), RBD (K417N, aa: 319–541), RBD (E484K, aa: 319–541), RBD (N501Y, aa: 319–541), and RBD‐Mut (K417N, E484K, N501Y, aa: 319–541) with His fragment, all of the recombinant proteins were purchased from Sino Biological. The purity of recombinant protein S1‐WT, S1‐Mut, RBD‐WT, RBD (K417N), RBD (E484K), RBD (N501Y), and RBD‐Mut was more than 90%, 90%, 95 %, 87%, 90%, 95%, and 90%, respectively. The endotoxin of these recombinant proteins was lower than 1.0EU per protein as determined by the LAL method.

The recombinant protein vaccines were prepared by mixing S1‐WT or S1‐Mut protein with AddaS03 adjuvant (AS03, InvivoGen, France) to be emulsifiable mixture. The bivalent vaccine was prepared with S1‐WT protein, S1‐Mut protein, and AS03 adjuvant.

We purchased female transgenic hACE2 mice with Institute of Cancer Research (ICR) background at 6–8 weeks (HFK bioscience company, China) for immunization. hACE2 mice received immunization by intramuscular injection of PBS, S1‐WT vaccine, S1‐Mut vaccine, and bivalent vaccine on days 0, 14, and 28, separately. Blood samples were collected on day 35 via eye socket vein, and sera was separated by centrifugation at 6000 rpm for 10 min, stored at ‐20℃ before using. All animal experiments have been approved by the Institutional Animal Care and Use Committee of Sichuan University (Chengdu, Sichuan, China).

### 4.4 Measurement of specific antibodies

The flat‐bottom 96‐well high binding plates (NUNC‐MaxiSorp, Thermo Fisher Scientific) were coated with 1 μg/ml antigen (S1‐WT, S1‐Mut, RBD‐WT, and RBD‐Mut) which was dissolved in carbonate coating buffer (50 mM, pH 9.6) per well at 4℃ overnight. Each well was washed three times with PBS containing 0.1% Tween (PBST), and blocked with 1% BSA solution for 1 h at room temperature. We diluted mouse sera in wells consecutively, followed by incubation at 37℃ for 1 h and three‐time washing of the plate with PBST. Add diluted anti‐mouse horseradish peroxidase antibody (Southern Biotech, USA) to each well at room temperature. After 1‐h incubation, we used 200 μl PBST to wash the plate for five times. By adding 3,3′,5,5′‐tetramethyl biphenyl diamine substrate, the reaction was quenched with 50 μl/well of 1.0 M H_2_SO_4_ stopping solution, and the absorbance was measured by 450 nm on a microplate reader.

### 4.5 Blockade of RBD and mutated RBD binding to receptor ACE2

Cell receptor ACE2 binding of RBD or mutated RBD was performed as described previously.^27^ Briefly, 0.3 μg/ml of RBD‐Fc, RBD (K417N)‐His, RBD (E484K)‐His, RBD (N501Y)‐His or RBD (K417N, E484K, N501Y)‐His (RBD‐Mut) proteins was added to the 293T/ACE2 cells in the absence or presence with series of diluted mouse sera. After the incubation at room temperature for 30 min, we washed cells three times with PBS, and then stained with FITC‐labeled anti‐human IgG Fc or APC anti‐His secondary antibody (BioLegend, USA) at 4℃ for 30 min. The binding assay was detected by NovoCyte Flow Cytometer (ACEA Biosciences), and the results were analyzed with FlowJoV software.

### 4.6 Preparation of pseudovirus

The wild‐type and variants of SARS‐CoV‐2 luciferase‐expressing pseudoviruses were purchased from Genomeditech (China). In the neutralization assays, D614G, N501Y, or E484K represented pseudoviruses with a single mutation. The mutate sites of B.1.1.7, B.1.351, and P.1 pseudovirus were consistent with the description in Figure 1A. The luciferase system reagents consist of cell lysis solution, and substrate was from Promega (USA).

EGFP‐expressing pseudovirus of SARS‐CoV‐2 wild‐type and B.1.351 was performed as described previously.^27^ 293T cells were pre‐seeded at a density of about 1×10^6^ cells before the transfections. The SARS‐CoV‐2 pseudovirus system was produced by three plasmids, including EGFP‐expressing HIV‐1 genome (pLenti‐EGFP vector), psPAX2, and plasmids encoding codon‐optimized SARS‐CoV‐2 wild‐type S protein or B.1.351 mutant protein with the optimum ratio of three plasmids was 8:3:1. All the plasmids with 45 μg transfection reagent polyethyleneimine were added to 700 μl of opti‐MEM, and the incubation for 15 min at room temperature. Add the mixture to 293T cells to culture for 6 h. Then replace the supernatant with fresh medium. Pseudoviruses in the culture supernatants were harvested 48 h and 72 h after transfection, and supernatant was isolated by low‐speed centrifugation and stored at ‐80°C.

### 4.7 Pseudovirus neutralization assay

The pseudovirus neutralization was performed as described previously.^28^ Briefly, 293T/ACE2 cells were pre‐seeded in 96‐well plates with a density of 1×10^4^ cells per well and grown overnight. Luciferase‐expressing pseudovirus with or without mutation (D614G, B.1.1.7, B.1.351, P.1, N501Y, E484K) was pre‐incubated with serially diluted immune sera in 96‐well plates for 1 h at 37°C, respectively. Then added the mixture to 293T/ACE2 cells and followed by incubation for 48 h to express the reporter gene. The efficiency of viral entry was determined with a firefly luciferase assay. In brief, remove the supernatants of infected cells. Then add 50 μl PBS, 50 μl lysis reagent from a luciferase kit and luciferase substrate (Promega). Detect relative light units with a multi‐mode microplate reader (PerkinElmer).

For the EGFP‐expressing pseudovirus with or without mutation (WT, B.1.351) neutralization assay, the method is same as described above, and the number of EGFP‐positive cells was determined with fluorescent microscopy and flow cytometry.

### 4.8 Pathological evaluation of vital organs

The mice were euthanized on 42 days after first immunization for tissue processing. Vital organs such as lungs, hearts, livers, kidneys, and spleen were harvested and fixed in 10% phosphate‐buffered formalin for 7 days, embedded in paraffin and sectioned at 3 μm thickness. Sequential sections were stained with hematoxylin and eosin to assess pathology and organ damage. Stained slides were scanned with an upright microscope (Nikon).

### 4.9 Statistical analysis

The statistical analyses were performed using Prism software (GraphPad Prism 8.0). The comparisons between the two groups were performed using unpaired Student's *t*‐tests. Comparisons among multiple groups were performed using one‐way ANOVA followed by Tukey's multiple comparison post hoc test. *p* < 0.05 was considered significant.

## CONFLICT OF INTEREST

Guobo Shen, Li Yang, Jiong Li, Zhenling Wang, Wei Wang, Guangwen Lu, and Xiawei Wei are employees of WestVac Biopharma Co. Ltd. This work was supported by WestVac Biopharma Co. Ltd.

## ETHICS STATEMENT

All studies on animals were performed after approval by the Ethics Committee of Sichuan University in compliance with Guidelines for the Use and Care of Small Laboratory Animals.

## AUTHOR CONTRIBUTIONS

Xiawei Wei provided study concepts and designed the study. Zimin Chen, Wei Wang, and Guangwen Lu performed gene cloning, expression, and protein purification. Cai He and Hong Lei performed the vaccine formulation and vaccinations in mice and did the other experiments. Guobo Shen and Li Yang were involved with data acquisition. Xiangrong Song, Zhenling Wang, and Jiong Li were involved with quality control of data and algorithms. Xiawei Wei, Jingyun Yang, Xuemei He, and Weiqi Hong edited the manuscript.

## Data Availability

The data included in this study are available upon request from the corresponding author.

## References

[1] Ni Y , Alu A , Lei H , Wang Y , Wu M , Wei X . Immunological perspectives on the pathogenesis, diagnosis, prevention and treatment of COVID‐19. Mol Biomed. 2021;2(1). 10.1186/s43556-020-00015-y PMC7815329 34766001

[2] Plante JA , Mitchell BM , Plante KS , Debbink K , Weaver SC , Menachery VD . The variant gambit: COVID‐19's next move. Cell Host Microbe. 2021;29(4):508–515. 33789086 10.1016/j.chom.2021.02.020 PMC7919536

[3] Gu H , Chen Q , Yang G , et al. Adaptation of SARS‐CoV‐2 in BALB/c mice for testing vaccine efficacy. Science. 2020;369(6511):1603–1607. 32732280 10.1126/science.abc4730 PMC7574913

[4] Zhou P , Yang XL , Wang XG , et al. A pneumonia outbreak associated with a new coronavirus of probable bat origin. Nature. 2020;579(7798):270–273. 32015507 10.1038/s41586-020-2012-7 PMC7095418

[5] Graham BS . Rapid COVID‐19 vaccine development. Science. 2020;368(6494):945–946. 32385100 10.1126/science.abb8923

[6] Graham BS , Mascola JR , Fauci AS . Novel vaccine technologies: essential components of an adequate response to emerging viral diseases. JAMA. 2018;319(14):1431–1432. 29566112 10.1001/jama.2018.0345

[7] Thanh Le T , Andreadakis Z , Kumar A , et al. The COVID‐19 vaccine development landscape. Nat Rev Drug Discov. 2020;19(5):305–306. 32273591 10.1038/d41573-020-00073-5

[8] Yi C , Sun X , Ye J , et al. Key residues of the receptor binding motif in the spike protein of SARS‐CoV‐2 that interact with ACE2 and neutralizing antibodies. Cell Mol Immunol. 2020;17(6):621–630. 32415260 10.1038/s41423-020-0458-z PMC7227451

[9] Wang P , Nair MS , Liu L , et al. Antibody resistance of SARS‐CoV‐2 variants B.1.351 and B.1.1.7. 2021;593:130–135. 10.1038/s41586-021-03398-2 33684923

[10] Tegally H , Wilkinson E , Giovanetti M , et al. Detection of a SARS‐CoV‐2 variant of concern in South Africa. Nature. 2021;592:438–443. 33690265 10.1038/s41586-021-03402-9

[11] Greaney AJ , Loes AN , Crawford KHD , et al. Comprehensive mapping of mutations in the SARS‐CoV‐2 receptor‐binding domain that affect recognition by polyclonal human plasma antibodies. Cell Host Microbe. 2021;29(3):463–476.e6. 33592168 10.1016/j.chom.2021.02.003 PMC7869748

[12] Cele S , Gazy I , Jackson L , et al. Escape of SARS‐CoV‐2 501Y.V2 from neutralization by convalescent plasma. Nature. 2021;593:142–146. 33780970 10.1038/s41586-021-03471-w PMC9867906

[13] Wibmer CK , Ayres F , Hermanus T , et al. SARS‐CoV‐2 501Y.V2 escapes neutralization by South African COVID‐19 donor plasma. Nat Med. 2021;27(4):622–625. 33654292 10.1038/s41591-021-01285-x

[14] Madhi SA , Baillie V , Cutland CL , et al. Efficacy of the ChAdOx1 nCoV‐19 covid‐19 vaccine against the b.1.351 variant. N Engl J Med. 2021. 10.1056/NEJMoa2102214. PMC7993410 33725432

[15] Portolano N , Watson PJ , Fairall L , et al. Recombinant protein expression for structural biology in HEK 293F suspension cells: a novel and accessible approach. J Vis Exp. 2014;e51897. 25349981 10.3791/51897 PMC4420617

[16] Zhu J . Mammalian cell protein expression for biopharmaceutical production. Biotechnol Adv. 2012;30(5):1158–1170. 21968146 10.1016/j.biotechadv.2011.08.022

[17] Hoffmann M , Arora P , Gross R , et al. SARS‐CoV‐2 variants B.1.351 and P.1 escape from neutralizing antibodies. Cell. 2021;184(9):2384–2393.e12. 33794143 10.1016/j.cell.2021.03.036 PMC7980144

[18] Zhou D , Dejnirattisai W , Supasa P , et al. Evidence of escape of SARS‐CoV‐2 variant B.1.351 from natural and vaccine‐induced sera. Cell. 2021;184(9):2348–2361.e6. 33730597 10.1016/j.cell.2021.02.037 PMC7901269

[19] Edara VV , Norwood C , Floyd K , et al. Infection‐ and vaccine‐induced antibody binding and neutralization of the B.1.351 SARS‐CoV‐2 variant. Cell Host Microbe. 2021;29(4):516–521. 33798491 10.1016/j.chom.2021.03.009 PMC7980225

[20] Fischer RJ , van Doremalen N , Adney DR , et al. ChAdOx1 nCoV‐19 (AZD1222) protects hamsters against SARS‐CoV‐2 B.1.351 and B.1.1.7 disease. bioRxiv. 2021. 10.1101/2021.03.11.435000. PMC8497486 34620866

[21] Focosi D , Maggi F . Neutralising antibody escape of SARS‐CoV‐2 spike protein: risk assessment for antibody‐based Covid‐19 therapeutics and vaccines. Rev Med Virol. 2021. 10.1002/rmv.2231. PMC8250244 33724631

[22] Jangra S , Ye C , Rathnasinghe R , et al. SARS‐CoV‐2 spike E484K mutation reduces antibody neutralisation. Lancet Microbe. 2021. 10.1016/S2666-5247(21)00068-9. PMC8026167 33846703

[23] Andreano E , Piccini G , Licastro D , et al. SARS‐CoV‐2 escape in vitro from a highly neutralizing COVID‐19 convalescent plasma. bioRxiv. 2020. 10.1101/2020.12.28.424451. PMC8433494 34417349

[24] Park BS , Lee N . A bivalent fusion vaccine composed of recombinant Apx proteins shows strong protection against Actinobacillus pleuroneumoniae serovar 1 and 2 in a mouse model. Pathog Dis. 2019;77(2):ftz020. 30939190 10.1093/femspd/ftz020

[25] Issaro N , Wu F , Weng L , et al. Induction of immune responses by a novel recombinant fusion protein of enterovirus A71 in BALB/c mice. Mol Immunol. 2019;105:1–8. 30465931 10.1016/j.molimm.2018.09.018

[26] Arukha AP , Minhas V , Shrestha A , Gupta SK . Contraceptive efficacy of recombinant fusion protein comprising zona pellucida glycoprotein‐3 fragment and gonadotropin releasing hormone. J Reprod Immunol. 2016;114:18–26. 26859695 10.1016/j.jri.2016.01.004

[27] Yang J , Wang W , Chen Z , et al. A vaccine targeting the RBD of the S protein of SARS‐CoV‐2 induces protective immunity. Nature. 2020;586(7830):572–577. 32726802 10.1038/s41586-020-2599-8

[28] Lei H , Alu A , Yang J , et al. Cationic nanocarriers as potent adjuvants for recombinant S‐RBD vaccine of SARS‐CoV‐2. Signal Transduct Target Ther. 2020;5(1):291. 33311439 10.1038/s41392-020-00434-x PMC7729145

